# Revisiting the Semantic Severity of Anxiety and Depression: Computational Linguistic Study of Normalization and Pathologization

**DOI:** 10.2196/73950

**Published:** 2025-07-22

**Authors:** Vojtech Pisl, Ana-Maria Bucur, Ioana R Podina

**Affiliations:** 1 Department of Psychiatry, Faculty of Medicine in Pilsen, Charles University, Alej Svobody 76, Pilsen, 323 00, Czech Republic, 420 797727175; 2 National Institute of Mental Health, Klecany, Czech Republic; 3 Interdisciplinary School of Doctoral Studies, University of Bucharest, Bucharest, Romania; 4 Laboratory of Cognitive Clinical Sciences, University of Bucharest, Bucharest, Romania; 5 MINDCARE FOR ALL Association, Bucharest, Romania

**Keywords:** psychiatrization, concept creep, prevalence inflation, mental health discourse, depression, anxiety, semantic severity, pathologization, normalization, trauma

## Abstract

**Background:**

Psychiatrization may contribute to the deterioration of public mental health observed in recent decades. The cultural aspects of psychiatrization can be understood as a form of concept creep (progressive expansion) of mental health terminology. Over time, concepts of psychopathology have expanded to encompass a broader range of human experiences, potentially diluting their meaning. Accordingly, previous research has shown a gradual decline in the semantic severity of the word trauma. However, the semantic severity of anxiety and depression has been increasing over time.

**Objective:**

This study aims to replicate and explain the increases in semantic severity of anxiety and depression by distinguishing between disorder constructs (clinical terms) and lay emotional constructs (everyday emotional terms) and assessing how their semantic severity changes over time. Additionally, we investigate whether mental health discourse and the broader context in which these terms appear influence these changes.

**Methods:**

We analyzed the semantic severity of anxiety, depression, and trauma using leading paragraphs from 4.7 million *New York Times* articles (1970-2023). We extended this analysis to broader disorder constructs (both generic terms, such as mental illness, and specific terms, such as schizophrenia) and lay emotional constructs (eg, sad and worried). A word2vec model was used to estimate the degree to which these terms appeared in mental health–related contexts, and a Mental Health Index was developed to quantify shifts in discourse. Regression analyses were conducted to assess whether changes in semantic severity were influenced by time and context.

**Results:**

The semantic severity of depression increased significantly (*τ*=0.35; *P*<.001), while anxiety (*τ*=0.08; *P*=.42) and trauma (*τ*=0.10; *P*=.33) showed no significant change. However, when controlling for context, severity was consistently higher in mental health–related contexts, and the effect of time became nonsignificant. For specific mental disorder constructs (eg, schizophrenia), semantic severity decreased over time, whereas generic disorder terms (eg, mental illness) remained stable. Lay emotional constructs became increasingly associated with mental health discourse but showed no clear severity trend.

**Conclusions:**

The increasing semantic severity of depression appears to be driven by its growing presence in mental health discourse rather than an inherent shift in meaning. The declining severity of specific, but not generic disorder constructs suggests that the overall representation of mental disorders remains severe, despite its expansion to less serious experiences. Meanwhile, ordinary emotions such as sadness and fear are increasingly discussed in mental health contexts. These trends highlight the evolving cultural framing of mental health and suggest that psychiatrization is shaping public perceptions of emotional experiences.

## Introduction

### Background

Many potential explanations have been proposed for the deterioration of population mental health discussed in recent decades. However, increases in epidemiological indicators may also stem from factors beyond true prevalence, such as a growing tendency to interpret and diagnose human experiences as instances of psychopathology [1-4]. According to the prevalence inflation hypothesis, these cultural shifts may improve the detection of mental disorders and reduce stigmatization but also disempower individuals and facilitate overdiagnosis and overmedication [5,6], triggering a self-perpetuating cycle [7]. High prevalence estimates motivate mental health campaigns that, in turn, encourage the interpretation of adversities as psychopathology, further inflating the observed prevalence. This spiral may have harmful consequences because perceiving adverse experiences as signs of psychopathology heightens their perceived severity, intensifying adversities by drawing attention to them and causing additional distress [7-9].

Psychiatrization refers to the increasing effect of psychiatric institutions, knowledge, and practices over more individuals and more areas of life [5]. Due to interactions between its material and ideological aspects, objective problems such as increasing consumption of psychotropics, overfilled clinics, or worsening epidemiological indicators may result from subtle cultural changes, such as the infusion of psychiatric terminology into everyday language or the overinterpretation of nonclinical problems as psychopathology [5]. These cultural aspects of psychiatrization may be part of a wider cultural shift proposed by the concept creep theory (CCT) [6,10,11]. The CCT documents an increase in the tendency to perceive various human experiences as harmful and a gradual semantic expansion of harm-related concepts since at least the 1970s [6,11]. Besides pioneering qualitative research [10] and observations of sharp increases in the use of psychological terms [12-15], the CCT is supported by studies using computational linguistics. The paradigm detects cultural trends in large collections of texts published across multiple decades using linguistic algorithms (see [16] for a methodological overview related to mental health and [17-19] for notable examples).

The CCT recognizes horizontal and vertical concept creep, referring to the extension of a word’s meaning to novel situations and its downward expansion to encompass less severe phenomena, respectively [11]. The latter can be measured using semantic severity: the emotional intensity and negativity associated with a specific word’s context. Words used in emotionally intense and negative contexts have higher severity, suggesting an association with more severe or distressing meanings. For instance, if the term catastrophe denotes a more serious problem than a hiccup, then catastrophe is discussed in more intense and negative contexts, resulting in higher semantic severity. Similarly, if a problem’s perceived severity increases over time, it is discussed in increasingly emotional and negative contexts, leading to a rise in the semantic severity of the corresponding word.

According to the CCT, the expansion of harm-related concepts should lead to their trivialization, reflected by a gradual reduction in semantic severity. For instance, if the term trauma is applied to a broader range of experiences [12,20], the average intensity of an experience described by the word trauma should be reduced, diluting the meaning of the word. An analysis of 871,340 psychology abstracts confirmed that the semantic severity of trauma has declined since the 1970s [20]. Building on these findings, Xiao et al [14] analyzed the severity of anxiety and depression in academic abstracts and a diverse collection of American texts, expecting a similar decrease in documenting vertical concept creep. However, the severity increased for both terms and across both text collections, indicating that anxiety and depression are becoming more pathologized rather than normalized.

### This Study

Previous research has shown that while the semantic severity of trauma has decreased, the severity of anxiety and depression has increased over time. However, the reasons behind these shifts remain unclear. This study aims to replicate these trends and explain them by studying whether these terms are becoming more severe due to their increasing presence in mental health discourse (eg, discussions of diagnosis, treatment, and psychiatric conditions), compared to other discourse contexts, such as economic language (eg, “economic depression”) or general everyday language (eg, “feeling anxious about an exam”).

Furthermore, we examine whether the changes in severity differ among specific kinds of constructs: (1) disorder-specific constructs (clinical terms referring to specific psychiatric disorders, eg, schizophrenia and bipolar disorder), (2) disorder-general constructs (broader terms referring to psychiatric disorders in general, eg, mental illness and psychiatric disorder), and (3) lay emotional constructs (terms describing general emotional states related to depression and anxiety, eg, sad and worried).

To achieve this, we conduct a large-scale corpus analysis of 4.7 million articles from *The New York Times* (1970‐2023) and use word2vec models to track how the meanings of these terms evolve across different contexts. This approach enables us to determine whether the semantic severity shifts are consistent or whether they vary depending on the construct and context in which the terms appear, in 3 separate analyses.

First, we focus on the semantic severity of anxiety, depression, and trauma*.* The frequency of the words depression and anxiety has quadrupled since the 1970s, and their use is increasingly linked to psychiatric disorder constructs [14]. Thus, the semantic severity of these terms might be rising because the terms more often refer to mental health constructs, which are intrinsically more serious, even if the perceived severity of these constructs had remained stable. For example, more frequent focus on psychological depressions compared to meteorological depressions may lead to an increase in the semantic intensity of the term depression, assuming that the mental health discourse is associated with more negative emotional valence than the meteorological one. We expect these words to be increasingly used in mental health contexts and their semantic severity to be increasing, replicating the previous results on a similar corpus [14]. Furthermore, we expect this increase to be explained by their increasing use in the mental health discourse.

Second, we focus on the semantic severity of nosological categories (further referred to as disorder constructs). Even within the mental health discourse, the words depression and anxiety may refer to either a disorder (eg, major depressive disorder and generalized anxiety disorder) or the underlying human experience (eg, feeling depressed or anxious). This may affect the observed time trends in the severity of these words. The semantic severity of depression and anxiety may have increased because these words are increasingly used to refer to clinical constructs rather than ordinary feelings. We measure the time trends in the semantic severity of 2 sets of words referring to mental disorders but not to the underlying human experiences: the disorder-specific and disorder-general constructs. We expect to observe a decrease in severity due to vertical concept creep. Furthermore, we expect this decrease to remain present even after controlling for the context of these words (ie, the extent to which the context refers to mental health).

Third, we focus on the terms referring to human experiences but not disorders (further referred to as lay emotional constructs). Because of the gradual psychiatrization of emotionally challenging experiences [1,21-23], we expect their semantic severity to be increasing. Furthermore, we expect their use to become more common in mental health contexts over time, reflecting an increasing tendency to interpret these experiences as psychiatric symptoms [7,24].

## Methods

### Corpus and Preprocessing

The leading paragraphs of *New York Times* articles (hereafter referred to as NYT corpus) published between 1970 and 2023 were retrieved from the New York Times Article Corpus [25], comprising 111,088,226 words from 4,748,684 articles. The corpus was lemmatized using the SpaCy library, stripped of nonwords, stop-words, and the term “Great Depression,” following the procedure used by Xiao et al [14].

### Ethical Considerations

This study is based solely on linguistic corpus data and does not involve any human participants or personal data. Therefore, no ethics approval was requested.

### Semantic Severity

Semantic severity was measured using the procedure introduced by Baes et al [20], approximating the severity of the situation described by a keyword as the negative emotionality expressed by the words surrounding this keyword. The severity of each use of the keyword of interest is calculated as the mean of the sums of arousal and negative valence associated with its 10 collocates (5 words preceding and 5 following the token), with valence and arousal estimated using the norms based on ratings from over a million human raters developed by Warriner et al [26]. A higher semantic severity score indicates that a word appears in more emotionally intense and negative contexts, suggesting an association with more severe or distressing experiences. For example, if depression frequently appears alongside words like suicidal, suffering, or severe, its semantic severity will be high. Conversely, a lower semantic severity score suggests that the word is used in less emotionally intense and negative contexts, implying a broader or more neutral use.

### Mental Health Index

The Mental Health Index (MHI) estimates how strongly a text is related to mental health discourse. It was constructed using a word2vec model, which quantifies the meaning of individual words. First, we trained a Continuous Bag of Words word2vec model on the entire corpus using the *word2vec* package in R (R Foundation for Statistical Computing) [27]. Next, we created a mental health vector by averaging the vectors of words strongly associated with mental health (eg, therapy, psychiatry, and diagnosis; see Multimedia Appendix 1 for details). This vector was used as a reference point for identifying mental health–related language. To validate its accuracy, we checked which words were most similar to the mental health vector. Next, we calculated the MHI for each text as the mean cosine similarity of all its words to the mental health vector, measuring how closely a text’s language aligns with mental health discourse. Finally, we have examined the vocabulary of groups of texts with high versus low index. As expected, texts with a high MHI contained words strongly related to mental health, confirming that the method identifies mental health discourse (Tables S1 and S2 in Multimedia Appendix 1).

### Operationalization of the Disorder and Lay Emotional Constructs

The disorder constructs were represented by 2 sets of terms: a generic set and a specific set. The generic one included 57 synonyms of the term “mental disorder” (eg, “mental illness” and “psychiatric disorder”). The specific one comprised collocates of 190 monosemous terms referring to specific mental disorders (eg, “anxiety disorder” or “anorexia”) collected from all 5 versions of the *DSM* (*Diagnostic and Statistical Manual of Mental Disorders*) and complemented with other common terms referring to disorder constructs (eg, “endogenous depression” or “depressive illness”; see Multimedia Appendix 1 for details). All terms from each set were treated as synonyms and analyzed together, similarly to previous studies [15,19]. No weighting was applied.

The lay emotional constructs were represented by 8 words referring to feeling depressed and anxious. The words were selected from synonyms of “anxiety,” “anxious,” “depression,” and “depressed” listed in the Merriam-Webster Thesaurus [28] (see Multimedia Appendix 1 for details). Considering potential heterogeneity among these terms, each term was analyzed separately.

### Statistical Analysis

To determine whether semantic severity changes over time, we created a simple model regressing severity on the year of publication. To determine whether this change persists after belongingness to mental discourse or context is accounted for, we added the MHI to the model. The descriptions and results of alternative statistical procedures are reported in Multimedia Appendix 1.

Reported results refer to 2-tailed tests with no correction for repeated testing, with statistical significance set at *P*<.05. The analysis was conducted in R (version 4.2.2); the scripts are available at Open Science Framework [29].

## Results

### Severity Trends for Anxiety, Depression, and Trauma

There were 4325 occurrences of the term anxiety, 3832 of depression, and 1559 of trauma. The mean semantic severity was 7.88 (SD 0.09) for anxiety, 7.82 (SD 0.19) for depression, and 7.95 (SD 0.18) for trauma.

The linear model regressing severity on the year of publication, reported in Table 1, was statistically significant for depression, indicating an increase in severity over time, but not for anxiety or trauma. A more direct replication of the previous study [14], reported in Multimedia Appendix 1, shows a statistically significant positive trend for depression, while the trends for anxiety and trauma are positive but not significant.

**Table 1.** Linear regression of semantic severity on the year of publication and Mental Health Index (MHI) for *New York Times* articles containing the keywords depression, anxiety, and trauma.

Keyword and model		*F* test (*df*)	*P* value	Year		MHI	
				β	*P* value	β	*P* value
Depression							
	Severity ~ year	*20.12 (1, 3826)^a^*	*<.001*	*.004*	*<.001*	—^b^	—
	Severity ~ year + MHI	*66.68 (2, 3825)*	*<.001*	.002	.11	*1.97*	*<.001*
Anxiety							
	Severity ~ year	0.29 (1, 4321)	.59	—	—	—	—
	Severity ~ year + MHI	*76.19 (2, 4320)*	*<.001*	<.001	.56	*2.64*	*<.001*
Trauma							
	Severity ~ year	0.20 (1, 1557)	.65	—	—	—	—
	Severity ~ year + MHI	*5.58 (2, 1556)*	*.003*	<.001	.71	*1.27*	*<.001*

a Values in italics format indicate statistically significant results.

b Not applicable.

When MHI was added to the models, all models were significant, revealing a positive relationship between semantic severity and MHI but not between severity and the year of publication.

These results are consistent with a sensitivity analysis splitting the sample into “mental health,” “economic,” and “remaining” texts (see Multimedia Appendix 1). Across all keywords, severity was higher for mental health texts compared to economic or remaining texts. Severity was unrelated to publication year when the analysis was conducted only for mental health or only for economic texts.

### Severity Trends for Mental Disorder Constructs

We identified 2159 occurrences of the generic terms (mean severity 7.93, SD 0.91) and 2975 of the specific terms (mean severity 7.77, SD 0.87). In the linear models reported in Table 2, severity was decreasing with time for specific terms but not for generic terms. When MHI was added to the equations, MHI predicted higher severity, and the year of publication predicted lower severity for specific terms. For generic terms, MHI predicted higher severity, while the year of publication had no effect.

**Table 2.** Linear regression models predicting the semantic severity of generic and specific mental disorder terms from the year of publication and the Mental Health Index (MHI).

Keywords and model		*F* test (*df*)	*P* value	Year		MHI	
				β	*P* value	β	*P* value
Generic							
	Severity ~ year	0.42 (1, 2157)	.52	—^a^	—	—	—
	Severity ~ year + MHI	*11.28 (2, 2156)^b^*	*<.001*	—	.74	*1.08*	*<.001*
Specific							
	Severity ~ year	*7.42 (1, 2946)*	*.006*	*−.004*	*.006*	—	—
	Severity ~ year + MHI	*9.54 (2, 2964)*	*<.001*	*−.004*	*.002*	*.67*	*.001*

a Not applicable.

b Values in italics format indicate statistically significant results.

A post-hoc analysis reported in Multimedia Appendix 1 revealed no systematic change in semantic severity when the time trends were examined for single generic or specific keywords (of 19 analyzed specific terms, the trend remained stable in 18 and increased in 1; of 8 analyzed generic terms, it remained stable in 6, increased in 1, and decreased in 1; only keywords with at least 30 occurrences were analyzed). The median Pearson correlation between the year of publication and severity was *r*=0.00 for specific terms and *r*=0.01 for generic terms.

### Severity Trends for Lay Emotional Constructs Related to Anxiety and Depression

There were 2228‐22,858 occurrences of the respective keywords (see Table S5 in Multimedia Appendix 1 for details). The association with mental health discourse was increasing over time for all 8 lay emotional terms, as reported in Table 3. The increase was particularly notable for the terms related to anxiety since the late 1990s to the early 2020s (Figure S4 in Multimedia Appendix 1). Later years of publication predicted lower severity for “unhappy” and “sorrow” and higher severity for “worry.”

**Table 3.** Regression models predicting the semantic severity of words describing anxious and sad feelings from the year of publication and Mental Health Index (MHI).

Keywords and model		*F* test (*df*)	*P* value	Year		MHI	
				β	*P* value	β	*P* value
Fear							
	Severity ~ year	1.40 (1, 23,567)	.24	—^a^	—	—	—
	Severity ~ year + MHI	*113.60 (2, 23,566)^b^*	*<.001*	—	*.77*	*1.93*	*<.001*
Worried							
	Severity ~ year	38.10 (1, 4145)	.05	—	—	—	—
	Severity ~ year + MHI	*44.12 (2, 4144)*	*<.001*	—	.40	*2.48*	*<.001*
Worry							
	Severity ~ year	*16.72 (1, 11,647)*	*<.001*	*.002*	*<.001*	—	—
	Severity ~ year + MHI	*114.10 (2, 11,646)*	*<.001*	*.001*	*.01*	*2.22*	*<.001*
Grief							
	Severity ~ year	0.06 (1, 2344)	.80	—	—	—	—
	Severity ~ year + MHI	*19.10 (2, 2343)*	*<.001*	—	.60	*3.09*	*<.001*
Sad							
	Severity ~ year	0.00 (1, 4881)	.99	—	—	—	—
	Severity ~ year + MHI	*60.05 (2, 4880)*	*<.001*	—	.59	*3.30*	*<.001*
Sadness							
	Severity ~ year	0.00 (1, 2643)	.96	—	—	—	—
	Severity ~ year + MHI	*5.96 (2, 2642)*	*.002*	—	.79	*1.54*	*<.001*
Sorrow							
	Severity ~ year	*25.17 (1, 6774)*	*<.001*	*−.004*	*<.001*	—	—
	Severity ~ year + MHI	*235.30 (2, 6773)*	*<.001*	*−.004*	*<.001*	*6.08*	*<.001*
Unhappy							
	Severity ~ year	3.81 (1, 2574)	.05	—	—	—	—
	Severity ~ year + MHI	*12.61 (2, 2573)*	*<.001*	*−.002*	*.02*	*1.64*	*<.001*

a Not applicable.

b Values in italics format indicate statistically significant results.

## Discussion

### Principal Findings

This study aimed to replicate and explain previously observed increases in the semantic severity of psychiatric terms while distinguishing between different types of constructs and discourse contexts. Our analysis of articles published in the *New York Times* between 1970 and 2023 supports prior research, showing that the severity of depression has increased over time, while trends for anxiety and trauma were less pronounced. Below, we discuss these findings in detail, along with additional insights into how discourse context and construct distinctions shape semantic severity and how these changes may affect public mental health.

### Pathologization of Anxiety, Depression, and Trauma

Our analysis revealed an increasing trend in the semantic severity of the word depression and positive, yet, statistically insignificant trends for anxiety and trauma. However, these shifts were largely explained by the increasing presence of these terms in mental health contexts.

Our results are consistent with findings of a large increase in the severity of depression and a substantially smaller increase in anxiety documented in general American English and academic abstracts [14]. Our null result related to anxiety may be due to lower statistical power. While the NYT corpus is more homogeneous, this may have not fully compensated for its smaller size compared to the corpora used in the previous study. Our null results regarding trauma contrast with a large decrease in semantic severity documented in academic abstracts in psychology [20]. While this may also be due to insufficient power, it may alternatively indicate differences between the academic and mass media documents. For example, trauma may be increasingly used loosely in subjective, expressive descriptions of harm rather than as a technical term constrained by a precise definition.

The terms of interest exhibited higher semantic severity in texts related to mental health compared to economic or other texts. Moreover, when we limited our analysis to texts referring to mental health, the semantic severity of depression was no longer increasing over time. Finally, more recent articles were more often related to mental health than the less recent ones. This is consistent with the observation that the diagnostic explanation of human hardships is gaining popularity at the expense of other explanatory frameworks [5,30].

Thus, the increase in semantic severity [14] replicated by us may be, partly or fully, explained by the growing number of texts referring to mental health. The increasing semantic severity of depression and other psychological terms may not signify a shift from “depression is bad” to “depression is terrible.” Rather, it may reflect shifting from diverse discussions about depression in heterogeneous contexts to rather stereotypical contents crafted to promote mental health awareness and facilitate help-seeking. These goals are undoubtedly relevant. Yet, as these efforts expand, public representations of mental health difficulties may become increasingly stereotypical, exaggerating the harm of even minor deviations from optimal well-being and reducing human suffering to prefabricated clinical categories while overlooking its social, existential, and ethical dimensions, as well as the respective sources of resilience.

### Missing Normalization of Generic Disorder Constructs

Our results revealed a decrease in the semantic severity of terms describing specific mental disorders (eg, “autism” and “anxiety disorder”) but not generic disorder terms (eg, “mental disorder” and “psychiatric illness”). Together, these results indicate that while less severe experiences are now being classified as mental disorders, the perceived severity of mental disorders overall does not decrease.

Three mechanisms may explain the decreasing severity of specific disorder terms. First, the disorders may be increasingly referring to less severe circumstances, akin to the vertical concept creep [10,11]. A post-hoc analysis, however, did not reveal any decrease in severity for individual disorder constructs, which does not support this explanation. Additionally, recent meta-analysis found no evidence of vertical concept creep in formal psychiatric diagnoses based on different versions of the *DSM* [31]. While formal diagnoses certainly differ from their public representations, the 2 mutually influence one another [5,32], making their long-term trends likely to converge over time. Second, novel disorder constructs introduced since 1970 (eg, attention-deficit/hyperactivity disorder or Asperger syndrome) may have lower semantic severity than the more traditional ones (eg, schizophrenia or autism), reducing the mean severity across all constructs in the more recent period. This explanation aligns with horizontal concept creep [10,11]. Third, growing public interest in mental health may lead the mass media to focus more on less severe mental disorders, as these are often more personally relevant to their audience. This supports observations that popular mental health discourse tends to underrepresent the struggles of individuals with severe mental disorders [33].

The severity of the generic terms referring to the umbrella category “mental disorders,” however, remained unchanged. Together, these 2 results may suggest that while novel and less severe experiences are newly classified as mental disorders, the mental representation of psychiatric disorders as a category does not change. Indeed, human cognition represents the fuzzy categories used in daily life by prototypes rather than formal definitions [34,35]. This allows, for instance, categorizing an injured 3-legged animal as a dog while retaining the mental representation of dogs as 4-legged creatures. Analogically, when novel, less severe experiences are added to the category of mental disorders, the category itself may retain its original severity. This process may, however, have negative clinical consequences. Categorizing less disturbing experiences as disorders while retaining the original perceived severity of the whole category increases their perceived severity. The increased perceived severity may, in turn, become self-fulfilling, inflating epidemiological indicators and undermining public mental health [7,8,36].

### Pathologization of Lay Emotional Constructs

Ordinary words describing depressive or anxious feelings, such as “sad” or “worried,” were increasingly associated with mental health contexts, especially over the last 2 decades. This observation mirrors previous findings of increasing pathologization of words describing undesirable mental traits and states in the American English [37] and the Czech language [24]. These findings are consistent with the notions of increasing psychiatrization in general [1,5] and specifically with respect to depressive [22,23] and anxious [21] experiences.

The semantic severity of the lay emotional words remained stable, with an increasing but statistically insignificant median trend. This suggests that while these words are increasingly linked to mental health, their fundamental meaning remains stable. This is consistent with linguistic research showing that core category words are more resistant to meaning change than specialized terms [38,39]. These results do not fully align with findings of decreasing semantic severity of the words addiction, grief, stress, and worry (but not anger and distress) [37].

The comparison of our results with the previous study with a very similar design [37] sheds light on the precision of our findings. The wide, heterogeneous corpus of written American English used in the previous study should reflect the same cultural trends as the NYT corpus used by us. Yet, we observed an increase in the severity of the word worry, regardless of whether we controlled for mental health discourse, in contrast to the decrease observed in the previous study, which was also unaffected by controlling for proximity to the mental health discourse. Similarly, our data revealed a positive, albeit insignificant, trend for the word fear, in contrast to the negative relationship in the previous study. These contradictions suggest that the differences between corpora may be larger than the trends in semantic severity we aimed to detect, especially for lay words, which are changing less substantially than professional terminology. Moreover, the increase in the severity of worry contrasts with no change in the severity of worried in our data, suggesting that the selection of specific words may also substantially affect semantic severity. At the same time, the proximity to the mental health discourse was increasing consistently across all terms, aligning with the predominantly positive trends in pathologization observed by the previous study.

### Limitations

First, our study is limited by only including data from one specific source, the *New York Times*, which may be biased by phenomena such as changes in editorial policies, orientations to specific audiences, and specific ideological stances. In addition, our results may reflect broader trends in journalistic styles and societal sentiment nonspecific to mental health, as predicted by the CCT. Furthermore, our analyses may be affected by shifts in the frequency of use of the terms anxiety, depression, and trauma, when referring to mental health versus other constructs. While these trends are controlled for by including the MHI as a covariate, this procedure does not distinguish between using mental health terms in contexts unrelated to mental health versus referring to other than mental health constructs. Second, the strength and the complexity of the relationship between the semantic severity examined by our study and the perceived severity of the mental health constructs are yet to be examined. The corpus-based research of cultural shifts is a relatively new field. Despite its methodological advantages [16,40] and generally accepted studies tracking cultural developments of the last decades [15,17-19], its reliability and validity were not fully established. Third, media texts are, besides reflecting the cultural perception of disorder constructs, likely affected by intentional efforts to form culturally shared representations. Indeed, many campaigns attempt to promote specific views on mental struggles [7,33]. These efforts may have contradicting effects: destigmatization and prevention campaigns may aim for normalization but also increase pathologization by promoting psychopathological interpretations and emphasizing severity to promote help-seeking. These limitations may affect the generalizability of our findings, while our results may contribute to clarifying the precision of the method once more data are available.

### Conclusions

The semantic severity of the word depression increased, while the rising severity of anxiety and trauma remained statistically insignificant, replicating the previous findings. This trend appears to be driven by the growing prevalence of these terms in mental health discourse rather than an inherent shift in meaning. The severity of specific, but not generic disorder constructs declined. These findings suggest that while the concept of mental disorders is expanding to include less serious experiences, their overall representation remains severe. Meanwhile, ordinary emotions such as sadness and fear are increasingly discussed in mental health contexts, reflecting the psychiatrization of emotionally challenging experiences. Together, these results suggest that anxious and depressive feelings are gradually pathologized rather than normalized over the last decades, with an increasing variety of experiences being discussed as mental health problems. These trends highlight the evolving cultural framing of mental health and suggest that psychiatrization is shaping public perceptions of emotional experiences, potentially biasing epidemiological indicators and impacting public mental health.

## Supplementary material

10.2196/73950 Multimedia Appendix 1 Further details of the methods and the methods and results of supplementary and sensitivity analyses.

## Abbreviations

CCT: concept creep theory
*DSM*: *Diagnostic and Statistical Manual of Mental Disorders*
MHI: Mental Health Index
NYT corpus: the New York Times Corpus
