# Supplementary material for: Revisiting the Semantic Severity of Anxiety and Depression: Computational Linguistic Study of Normalization and Pathologization
Source: J Med Internet Res. 2025 Jul 22;27:e73950. doi: 10.2196/73950 (PMC12306585; doi:10.2196/73950)
Supplement: Multimedia Appendix 1 [file jmir-v27-e73950-s001.docx]

### Direct replication of Xiao et al 2023

#### Method

For a direct replication of the previous studies (Baes, Vylomova, et al., 2023; Xiao et al., 2023), only the time period from 1970 to 2018 was used and the semantic severity was analysed on annual basis rather than per document. Specifically, we have first calculated the annual semantic severity as the mean severity across all texts published in the given year. Correlational analysis was then used to quantify the time trends of these annual means. The Kendall’s correlation coefficient between these annual means and the year of publication was chosen as the primary measure to avoid outlier bias (particularly due to low number of corpus articles in the 1980 resulting in an anomaly visible on the Figure S1).

#### Results

There were 3,825 occurrences of the term *anxiety*, 3,588 of *depression,* and 1,319 of *trauma*. The mean semantic severity was 7.85 for anxiety, 7.88 for depression, and 7.94 for trauma.

The semantic severity was increasing over time for depression (τ = .35; *p* < .001) but not anxiety (τ = .08; *p* = .42) or trauma (τ = .10; *p* = .33; see Figure S1).


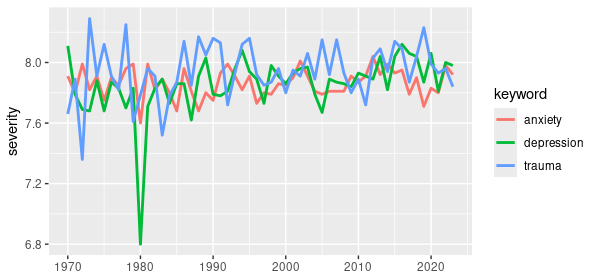


***Figure S1: The trends in the semantic severity of the collocates of depression, anxiety, and trauma in a corpus of leading paragraphs of articles published in the New York Times. The outlying value for depression in the 1980s is due to an irregularity in the corpus, only containing 3 occurrences of the word “depression” in that period. This does not affect the results, as the main results do not use the annual means and the supplementary results use non-parametric correlation.***

### Calculating mental and economic indices

#### The word2vec model

The Continuous Bag of Words (CBOW) model was used for its superior speed compared to alternatives. The file consisting of the leading paragraphs of the NYT articles had 804 MB. It took several hours to train the model with 10 iterations on a server with 16 cores, 120GB RAM, using the “Background jobs” in RStudio Server.

The lists of words related to economy and mental health used to define the economy and mental vectors were selected apriori by the first author and ChatGPT and remained unchanged after visual inspection has confirmed that the procedure successfully separated economic from mental topics.

**Economy**: economy market recession finance capital investment credit gold business investor

**Mental_health**: mental schizophrenia health disease drug therapy melancholia psychiatry psychology

The relationship of the economy and mental health vectors to economic and mental health vocabulary was confirmed by inspection of 20 terms with highest similarity to each vector reported below.

| **20 words most similar to the economic (left) and mental (right) vector** | | | | |
| --- | --- | --- | --- | --- |
| **rank** | **term** | **similarity** | **term** | **similarity** |
| 1 | investor | 0.656406 | schizophrenia | 0.6384102 |
| 2 | investment | 0.654073 | therapy | 0.628018 |
| 3 | market | 0.653034 | disease | 0.6077579 |
| 4 | economy | 0.650322 | mental | 0.5985041 |
| 5 | business | 0.612115 | patient | 0.5959414 |
| 6 | financial | 0.598116 | disorder | 0.5945442 |
| 7 | sector | 0.594213 | psychology | 0.5922743 |
| 8 | currency | 0.589386 | psychiatry | 0.5902483 |
| 9 | lender | 0.587978 | alcoholism | 0.5899431 |
| 10 | recession | 0.58496 | psychotherapy | 0.5875734 |
| 11 | portfolio | 0.5845 | neurological | 0.585459 |
| 12 | shortterm | 0.579155 | clinical | 0.5852367 |
| 13 | stock | 0.578665 | pathology | 0.5805285 |
| 14 | economic | 0.578645 | behavioral | 0.5776449 |
| 15 | debt | 0.576963 | dementia | 0.5766096 |
| 16 | profit | 0.576678 | infertility | 0.5764159 |
| 17 | dollar | 0.576493 | hypnosis | 0.5763835 |
| 18 | banking | 0.576332 | treatment | 0.5760499 |
| 19 | industry | 0.574933 | medicine | 0.576003 |
| 20 | invest | 0.574708 | epilepsy | 0.5758254 |

***Table S1 The words with highest similarity to the economic and mental vector, respectively.***

#### Most common collocates by context

***Table S2: 20 most common collocates along with their frequencies of the terms depression, anxiety, and trauma, in the whole dataset and its subsets.***

### Time trends in mental and economic indices

#### Method

The same as for the direct replication. The mean annual economic and mental health indices were calculated for every year across all articles published that year. An economic index was calculated and validated analogically to the mental health index.

#### Results

The mental health index was increasing over time for texts containing the word *depression* (τ = .72; *p* < .001) but not *anxiety* (τ = .19; *p* = .05) or *trauma* (τ = .17; *p* = .08). The economic index was decreasing for texts containing *depression* (τ = -.27; *p* = .006), *anxiety* (τ = -.32; *p* < .001), and *trauma* (τ = -.25; *p* = .01; see Figure S2).


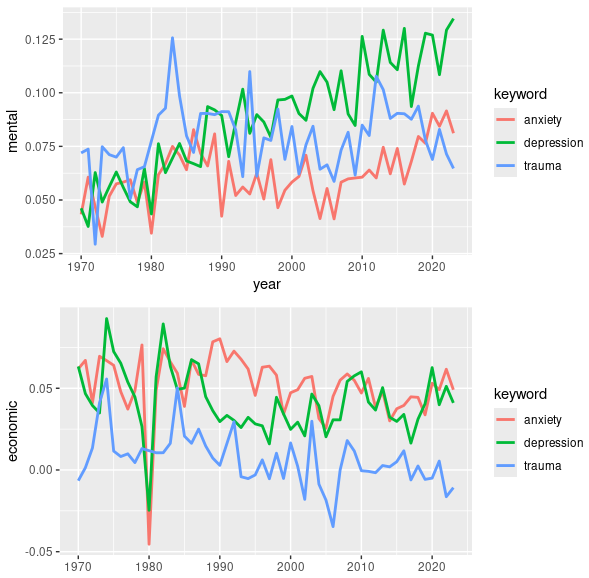


***Figure S2: lexical similarity to mental health (up) and economic (down) discourse for articles published in New York Times between 1970 and 2023 that contain the word anxiety, depression, and trauma, respectively.***

### Separate analyses for texts with mental health vs economy contexts

#### Methods

The same dataset and indices were used as in the Time Trends analysis. To compare the severity of documents referring to mental health and economic topic, we have labelled each text as “mental”, “economic”, or “remaining” based on their mental and economic indices. Each document with mental health index higher than economy index and higher than the 67^th^ percentile of all mental indices was classified as belonging to mental health discourse. Analogically, each document with the economy index higher than the mental index and higher than the 67^th^ percentile of all economy indices was classified as belonging to the economic discourse. All other documents were classified as “remaining”. The 67^th^ percentile was chosen arbitrarily, following manual inspection of the data. The categorization of the documents was confirmed by inspection of the most common collocates in each subset. ANOVA combined with Welsch t-tests was used to test group differences. Texts containing the word “trauma” were not analysed due to a lower sample size.

#### Results

The documents containing different keywords differed in the mental health (*F*(143,2)= 26.78, *p* < .001) as well as economic index (*F*(143,2)= 74.55, *p* < .001). The documents containing *trauma* (*t*(83.17) = 7.05, *p* < .001) or *depression* (*t*(69.23) = 6.64, *p* < .001) were more similar to the mental discourse than those containing *anxiety*, while the first two did not differ (*p* = .28). The economic index was higher in documents containing *anxiety* compared to those containing *depression* (*t*(95.96) = 2.75; *p* = .007)^[[1]](#footnote-1)^ which were of higher economic index than those containing *trauma* (*t*(91.82) = 9.09; *p* < .001).

Finally, we have separated the texts into three subsets by the mental and economic indices: “mental”, “economic”, and “remaining”. A two-way ANOVA (keyword × subset) confirmed a difference in severity between the “economic”, “mental” vs “remaining” subsets (*F*(2, 285) = 57.76; *p* <. 001) but not between texts containing the word anxiety versus depression (*F*(1,285) = 0.04; *p* = .84). The severity of the mental subset was higher than the economic one (*t*(175.84) = 5.88; *p* < .001) which itself had higher severity than the remaining subset (*t*(184.11) = 5.84; *p* < .001). No change in severity was observed in the mental subset (depression: τ = .02; *p* = .85; anxiety: τ = .07; *p* = .49) or in the economic subset (depression: τ = .02; *p* = .82; anxiety: τ = -.03; *p* = .84). In the remaining subset, we have observed an increasing severity of the collocates of depression (τ = .31; *p* = .001) but not anxiety (τ = .06; *p* = .58).

### Disorder and lay emotional constructs

#### Sets of terms: The disorder constructs

**Mental disorders – generic terms** were initially defined as all combinations of the words mental, psychiatric, psychological, emotional, behavioral, and mental health with words disorder, illness, disease, syndrome, disability, diagnosis, condition. The list was further extended with the help of ChatGPT and Google the terms “psychosis” and “neurosis”.

**Mental disorders – specific** were defined through lists prepared for another project. Terms referring uniquely to nosological categories (but not to feelings or symptoms – thus, “melancholia” was not included while “bulimia” was) were taken from the DSM-I to DSM-5 to cover the period from 1950s to 2020s. Terms were collected that clearly refer to a nosological construct. The terms were gained through three means:

1. Searching through DSM-I-5 for diagnostic labels
2. Adding similar terms clearly referring to the nosological construct known to the authors – like “depressive illness” or “endogenous / exogenous depression”
3. Consulting ICD 10 and internet and Wikipedia and ChatGPT for additional terms.

The list was developed in a way minimizing possible artefacts resulting from inclusion of only a part of a term comprising of multiple words. For instance, inclusion of the term “hyperactivity disorder” in the list would result in “attention” and “deficit” being counted as collocates when the whole term “attention deficit hyperactivity disorder” is used in a text. Thus, only the whole “attention deficit hyperactivity disorder” is included. At the same time, we attempted to extend the number of terms – so, we also include “attention hyperactivity disorder” or “hyperactivity attention disorder”. Similarly, we include “major depression” but not “major depressive” or “autistic illness” but not “autistic spectrum” etc.

#### Sets of terms: The lay emotional constructs

Our primary goal was to select terms that are predominantly used when referring to human experiences or emotions to mitigate the effect of different discourses. Our original intention was to include a wider variety of feelings that are often described as being pathologized by critical psychiatry (e.g., loneliness, suffering a loss, restlessness, shyness etc) – yet, we failed to find words that would primarily refer to these feelings in the NYT corpus (e.g., “lonely” often refers to geographical seclusion rather than feelings, “to stress” refers to emphasizing a matter rather than to cause tension, funds and options are “exhausted” more often than individuals).

Twelve words were initially selected that predominantly referred to human feelings in the corpus (e.g., “fear” was included as it predominantly refers to human emotion, but “concern” was not, because it is often used in other meanings): two sets of substantives referring to anxiety or to depression (e.g., sadness or worry) and two sets of respective adjectives (e.g., sad or worried). During the analysis, “unease” and “heartbroken” were then removed due to low sample size (436 and 209 occurrences, respectively) and “upset” and “nervous” were removed because their collocates referred to sports (upset victory) and nervous system rather than emotions.

**Lay emotional terms:** the words returned as the most relevant synonyms by the thesaurus (*The Merriam-Webster Thesaurus*, 2024) are listed below. Those that were manually removed due to polysemy or due to being present in more than one lists were removed (are crossed below):

**depression** sadness ~~melancholy~~ sorrow~~fulness~~ grief (melancholy removed due to overlap with DSM; sorrowfulness changed to sorrow due to low sample size of the former)

**anxiety** fear worry ~~concern~~ unease (concern removed as polysemous)

**depressed** unhappy sad heartbroken ~~miserable~~ ~~melancholy~~ ~~upset~~ ~~bad~~ ~~worried~~ ~~sorry~~ (removed as polysemous or repeating in different lists)

**anxious** worried nervous upset ~~uneasy~~ ~~apprehensive~~ ~~troubled~~ (removed as polysemous or repeating in different lists)

#### Collocates of the disorder constructs

***Table S3: 20 most common collocates of the generic and the specific mental disorder terms***

#### Collocates of the lay constructs

***Table S4: 20 most common collocates of the lay constructs***

#### Results: correlation analysis for disorder constructs

There was a decrease in severity of the specific disorder terms (τ = -.18; *p* = .06; Figure 4) but not the generic ones (τ = .07; *p* = .43) between 1970 and 2023. The mental index was unrelated to the year of publication for generic (τ = .04; *p* = .66) or specific (τ = .17; *p* = .07) terms.


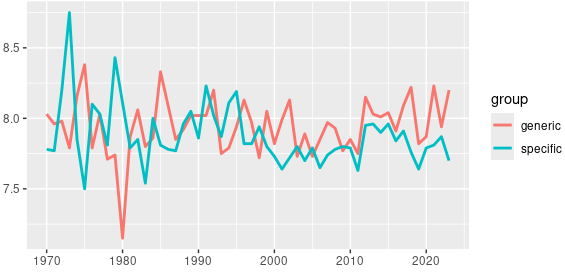


***Figure S3 Trends in semantic severity of generic and specific terms for mental disorders in fragments of articles published in the New York Times.***


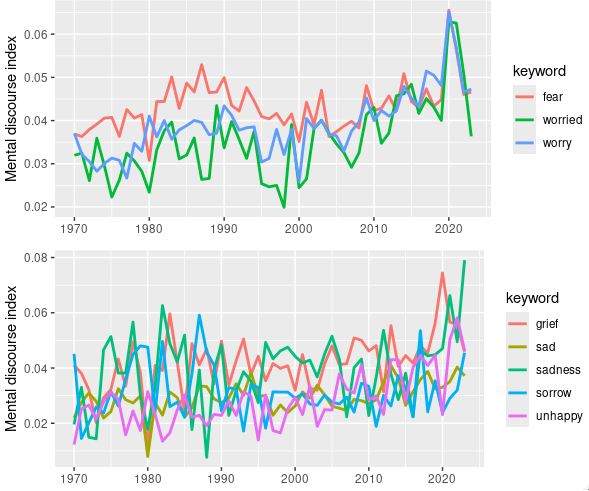


***Figure S4 Trends in proximity to mental health discourse of texts containing keywords related to anxiety (up) and depression (down) published in the New York Times between 1970 and 2023***

#### Results: correlation analysis for lay constructs

Of 8 words describing anxious or depressive feelings, severity has increased significantly only for “worry”, while all other words were showing statistically insignificant increase; see Figure 5 and Table 3 for details.

***Table S5 Trends in semantic severity and proximity to mental health discourse of words related to depression and anxiety published in the New York Times between 1970 and 2023.***

#### Disorder constructs: post hoc analysis by keyword

##### Method

Only keywords with at least 30 occurrences across the corpus were analysed. Their mean mental health index, severity score and year of publication is reported along with the Pearson correlation coefficient between the severity and year of publication for each specific keyword. Median correlation among all generic terms is reported as the primary result for generic terms; median correlation among all specific terms is reported as the primary result for specific term.

##### Results

As reported in the main text of the study, in one of 19 analysed specific terms the trend was increasing, in 18 others it remained stable; out of 8 analysed generic terms, 6 remained stable, 1 increased and 1 decreased. The median Pearson’s correlation between the year of publication and severity was *r* = -.00 for the specific terms and *r* = .01 for the generic ones. Detailed results are presented in the Table below. Notice that while “attention deficit hyperactivity disorder” is the only specific trend showing a significant change, the term “ADHD” does not follow this trend.

***Table S6 Descriptive statistics (n, mean mental index, mean severity and mean year of publication) and the correlation between severity and year of publication for generic and specific disorder terms with at least 30 occurrences. MHI = mental health index.***

1. While these results were contrary to our intuition that depression refers to economic matters more often than anxiety, inspection of the data confirmed that the word anxiety was indeed discussed in economic and geopolitical contexts more often than in reference to mental health (as documented by the most common collocates). Moreover, these results reflect the data after the term “Great Depression” was removed, decreasing the frequency of use of *depression* in economic contexts. [↑](#footnote-ref-1)
